# Supplementary material for: Nucleolar Dynamics During Oogenesis
Source: bioRxiv. 2026 May 20:2026.05.19.726235. Preprint. [Version 1] doi: 10.64898/2026.05.19.726235 (PMC13228488; doi:10.64898/2026.05.19.726235)
Supplement: 1 [file NIHPP2026.05.19.726235V1-supplement-1.pdf]

bioRxiv preprint doi: https://doi.org/10.64898/2026.05.19.726235; this version posted May 20, 2026. The copyright holder for this preprint (which was not certified by peer review) is the author/funder, who has granted bioRxiv a license to display the preprint in perpetuity. It is made available under a CC-BY 4.0 International license.

Li and McKown *et al.*, Fig. S1

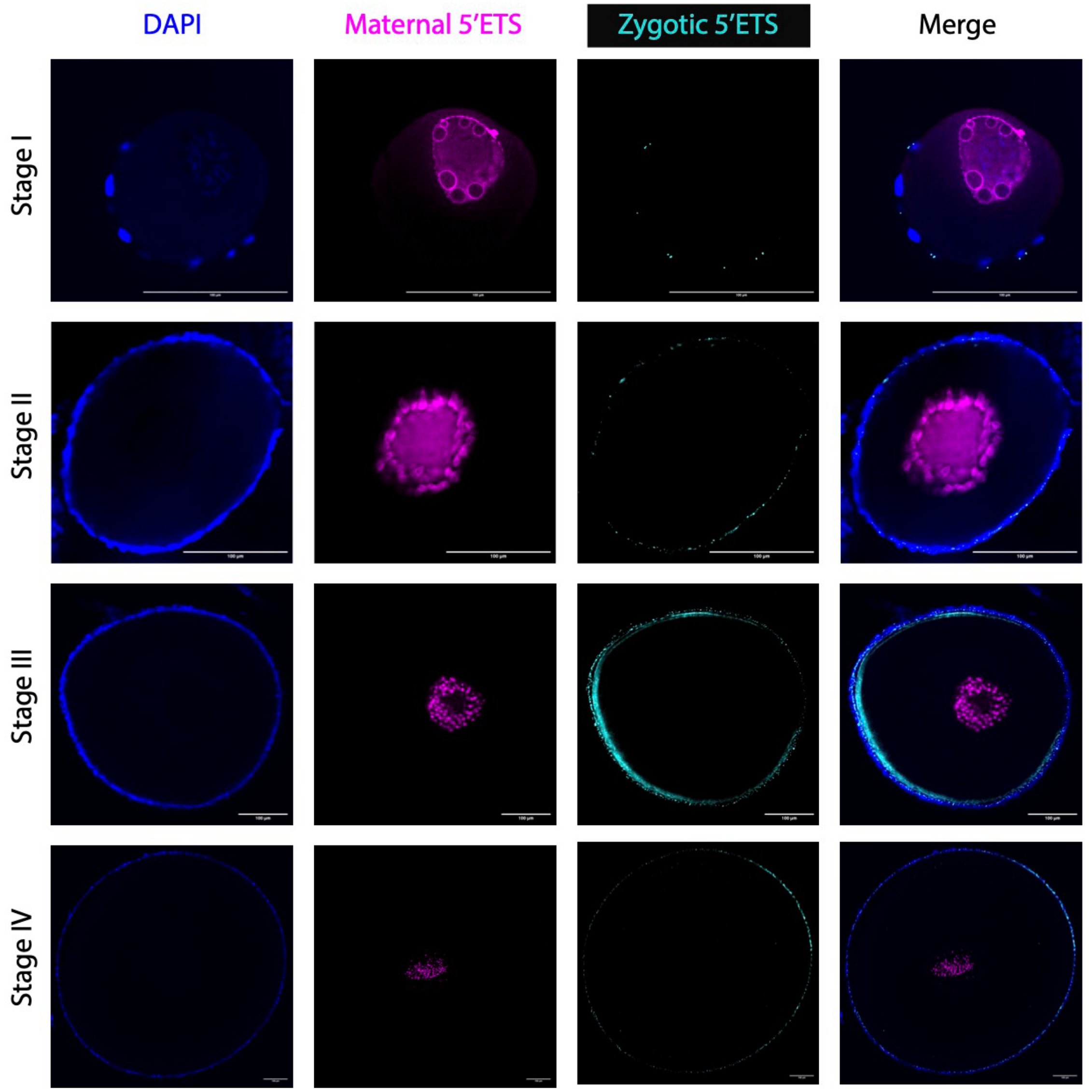

Fig. S1: rRNA synthesis in oocytes and surrounding somatic cells.
HCR RNA-FISH of nascent maternal (magenta) or zygotic (cyan) rRNA in whole-mount oocyte stages I-IV. Nuclei are stained with DAPI (blue). Note that zygotic rRNA synthesis occurs exclusively in surrounding granulosa cells, but not the developing oocyte. Scale bar: 100 µm.

bioRxiv preprint doi: https://doi.org/10.64898/2026.05.19.726235; this version posted May 20, 2026. The copyright holder for this preprint (which was not certified by peer review) is the author/funder, who has granted bioRxiv a license to display the preprint in perpetuity. It is made available under a CC-BY 4.0 International license.

Li and McKown *et al.*, Fig. S2

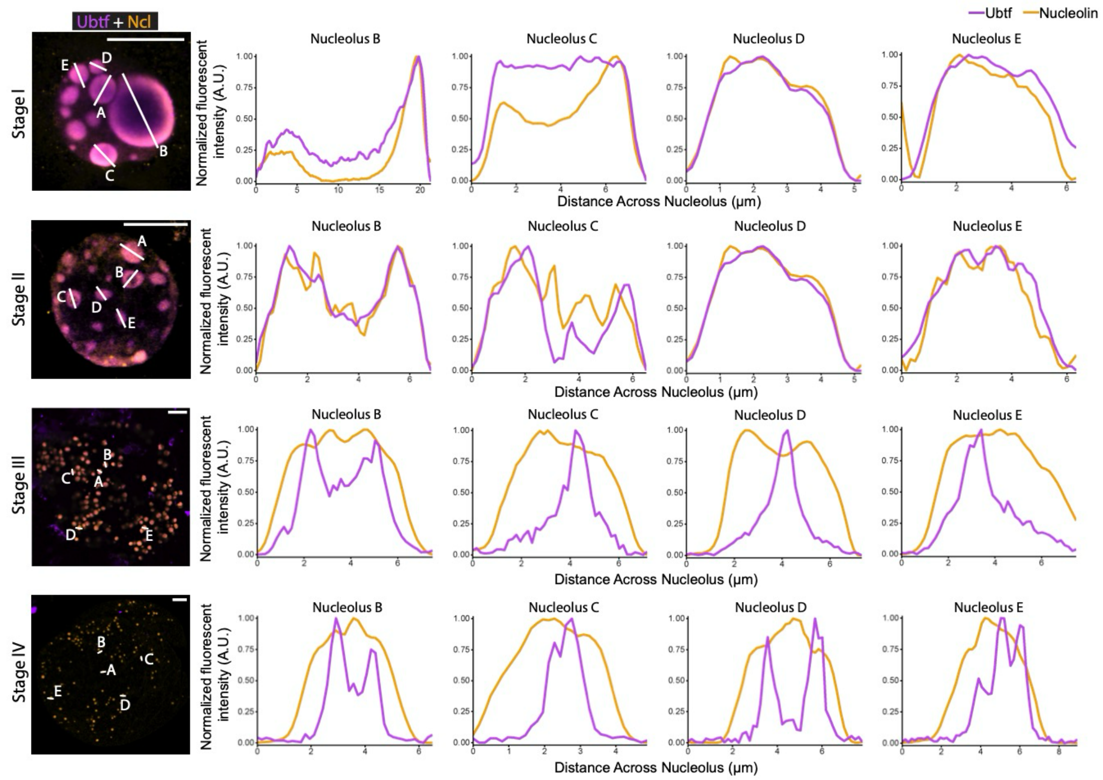

Fig. S2: Nucleolar layers in zebrafish oocytes exhibit distinct architectures. Quantitation of fluorescent intensity measurements of nucleoli in oocytes from Fig. 4B. Oocytes were immunostained for Ubtf (purple) and Nucleolin (orange). Nucleolus measured indicated by white line and labelled A-E. Brightness and contrast were adjusted for visualization. Intensities were normalized to minimum and maximum values. A. U., Arbitrary Units. Scale bar: 20 µm.

bioRxiv preprint doi: https://doi.org/10.64898/2026.05.19.726235; this version posted May 20, 2026. The copyright holder for this preprint (which was not certified by peer review) is the author/funder, who has granted bioRxiv a license to display the preprint in perpetuity. It is made available under a CC-BY 4.0 International license.

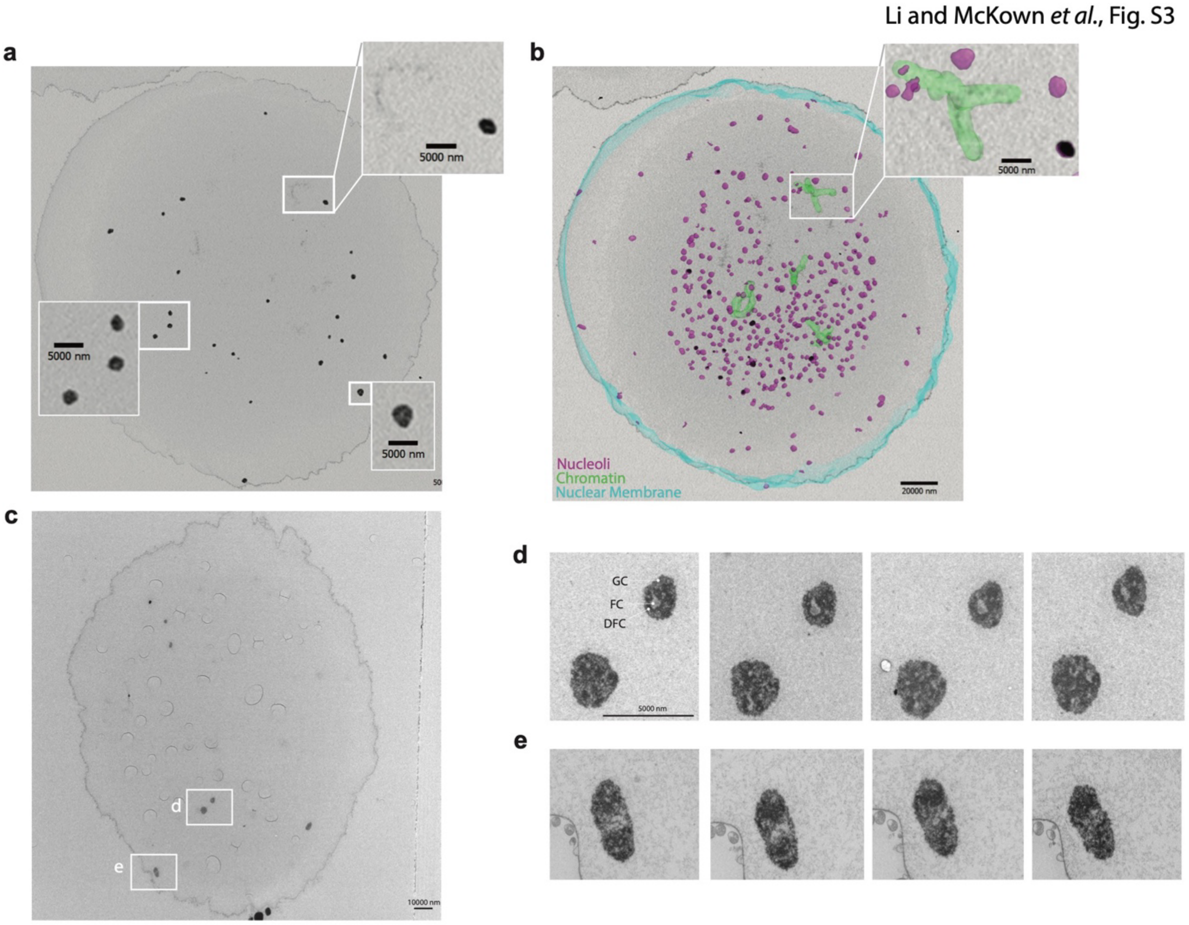

Fig. S3: Tripartite nucleoli in stage IV zebrafish oocytes.

(a) Array tomography of stage IV zebrafish nucleolus. Highlighted are nucleoli and condensed chromatin (white arrow). Scale bar: 5000 nm.

(b) 3D reconstruction of nuclear structures from 89 array tomography slices, 1 µm each. Highlighted are the nuclear membrane (blue), condensed chromatin (green), and nucleoli (magenta). Scale bar: 2000 nm.

(c) Overview of stage IV oocyte acquired with transmission electron microscopy (TEM). Scale bar: 10000 nm. Nucleoli imaged at higher resolution are highlighted.

(d, e) Serial images through selected nucleoli showing fibrillar center (FC) (hollow centers), surrounded by dense fibrillar component (DFC), and granular component (GC). Section thickness is 100 nm, scale bar: 5000 nm.

bioRxiv preprint doi: https://doi.org/10.64898/2026.05.19.726235; this version posted May 20, 2026. The copyright holder for this preprint (which was not certified by peer review) is the author/funder, who has granted bioRxiv a license to display the preprint in perpetuity. It is made available under a CC-BY 4.0 International license.

Li and McKown *et al.*, Fig. S4

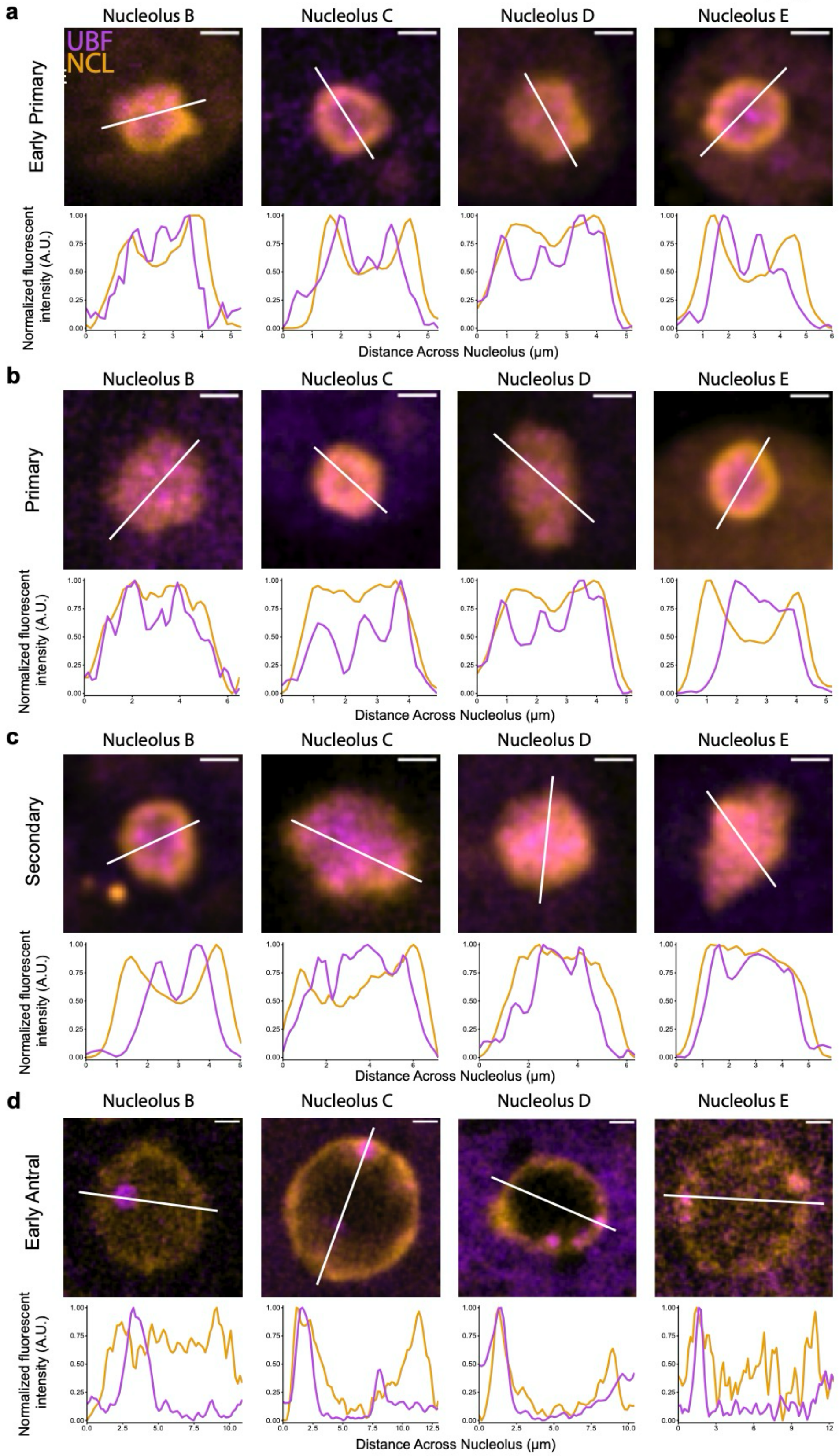

bioRxiv preprint doi: https://doi.org/10.64898/2026.05.19.726235; this version posted May 20, 2026. The copyright holder for this preprint (which was not certified by peer review) is the author/funder, who has granted bioRxiv a license to display the preprint in perpetuity. It is made available under a CC-BY 4.0 International license.

Fig. S4: Nucleolar layers in mouse oocytes exhibit distinct architectures.
(a-d) Quantitation of fluorescent intensities of UBF and Nucleolin across nucleoli of early primary (a), primary (b), secondary (c), and early antral (d) oocytes shown in images above. Intensities were normalized to minimum and maximum values. Early antral oocytes in panel d were imaged using a 500 ms longer exposure time for the 561 nm laser channel and 200 ms longer exposure time for the 647 nm laser channel relative to the other samples due to sample depth. A. U., Arbitrary Units. Scale bar: 2 μm.
